# Genome wide high density SNP-based linkage analysis of childhood absence epilepsy identifies a susceptibility locus on chromosome 3p23-p14

**DOI:** 10.1016/j.eplepsyres.2009.09.010

**Published:** 2009-12

**Authors:** Barry A. Chioza, Jean Aicardi, Harald Aschauer, Oebele Brouwer, Petra Callenbach, Athanasios Covanis, Joseph M. Dooley, Olivier Dulac, Martina Durner, Orvar Eeg-Olofsson, Martha Feucht, Mogens Laue Friis, Renzo Guerrini, Marianne Juel Kjeldsen, Rima Nabbout, Lina Nashef, Thomas Sander, Auli Sirén, Elaine Wirrell, Paul McKeigue, Robert Robinson, R. Mark Gardiner, Kate V. Everett

**Affiliations:** a Institute of Child Health, University College London, 30 Guilford Street, London WC1N 1EH, UK; b Hôpital Robert Debré, France; c Department of General Psychiatry, Medical University Vienna, Austria; d Department of Neurology, University Medical Centre Groningen, University of Groningen, The Netherlands; e Neurology Department, The Children's Hospital ‘Agia Sophia’, Greece; f Dalhousie University and IWK Health Centre, Canada; g Neuropaediatrics Department, Hôpital Necker Enfant Malades, France; h Division of Statistical Genetics, Columbia University, USA; i Department of Women's and Children's Health/Neuropaediatrics, Uppsala University, Sweden; j Department of Paediatrics, Medical University Vienna, Austria; k Department of Neurology, Sygehus Vestsjaelland, Denmark; l Division of Child Neurology and Psychiatry, University of Pisa, and IRCCS Fondazione Stella Maris, Italy; m Department of Neurology, Odense University Hospital, Denmark; n Neuroscience, King's College Hospital, UK; o Cologne Center for Genomics, University of Cologne, Cologne, Germany; p Epilepsy Genetics Group, Department of Neurology, Charité University Medicine, Humboldt University of Berlin, Germany; q Department of Paediatrics, Tampere University Hospital, Finland; r Division of Child and Adolescent Neurology, Mayo Clinic, USA; s Public Health Sciences Section, Division of Community Health Sciences, The University of Edinburgh Medical School, UK; t Paediatric Neurology, Great Ormond Street Hospital, UK

**Keywords:** Childhood absence epilepsy, Linkage, Association, Chromosome 3, *TRAK1*

## Abstract

Childhood absence epilepsy (CAE) is an idiopathic generalised epilepsy (IGE) characterised by typical absence seizures manifested by transitory loss of awareness with 2.5–4 Hz spike-wave complexes on ictal EEG. A genetic component to the aetiology is well recognised but the mechanism of inheritance and the genes involved are yet to be fully established.

A genome wide single nucleotide polymorphism (SNP)-based high density linkage scan was carried out using 41 nuclear pedigrees with at least two affected members. Multipoint parametric and non-parametric linkage analyses were performed using MERLIN 1.1.1 and a susceptibility locus was identified on chromosome 3p23-p14 (*Z*_mean_ = 3.9, *p* < 0.0001; HLOD = 3.3, *α* = 0.7). The linked region harbours the functional candidate genes *TRAK1* and *CACNA2D2*. Fine-mapping using a tagSNP approach demonstrated disease association with variants in *TRAK1*.

## Introduction

The absence epilepsies are a group of idiopathic generalised epilepsies (IGEs) which differ in their seizure frequency, age of onset and pattern of evolution. A typical absence seizure manifests as a transitory loss of awareness with 2.5–4 Hz spike-wave complexes on ictal EEG. Many patients also have generalised tonic–clonic seizures (GTCS), myoclonic seizures or febrile seizures in addition and a variety of ‘atypical’ absence seizures are recognised. The International League Against Epilepsy (ILAE) classification recognises a number of distinct absence epilepsy syndromes including childhood absence epilepsy (CAE), juvenile absence epilepsy (JAE), epilepsy with myoclonic absences, juvenile myoclonic epilepsy (JME) and eyelid myoclonia with absences as a seizure type (Engel, 2001). However, it is still unclear whether these syndromes represent a ‘biological continuum’ or distinct entities. Frequency of absence seizures per day is greater in CAE than JAE, and the occurrence of GTCS is greater in patients with JAE than CAE. However, there is evidence that CAE and JAE share a close genetic relationship allowing them to be considered as one phenotype in genetic studies (Berkovic et al., 1987; Marini et al., 2004).

Twin studies have demonstrated that the IGEs, including those in which absence seizures occur, have a significant heritability (Berkovic et al., 1998), with regards to both occurrence and type of seizure and syndrome with concordance rates for monozygotic twin pairs far higher than for dizygotic twin pairs (Kjeldsen et al., 2003). Absence epilepsies, along with the other common forms of IGE, show a complex pattern of inheritance. In keeping with other common genetic disorders, this is expected to result from the action of a few or many genes of small to moderate effect.

Genome-wide linkage analysis of IGE-multiplex families has demonstrated evidence for susceptibility loci on chromosomes 2q36, 3q26, and 14q23 (Sander et al., 2000). Furthermore, loci for three similar forms of absence epilepsy have been identified on chromosomes 8q24 (*ECA1*), 5q31.1 (*ECA2*) and 3q26 (*ECA3*) (Fong et al., 1998; Robinson et al., 2002; Sugimoto et al., 2000; Wallace et al., 2001). We have previously shown evidence for linkage and association to chromosome 16p12-p13.1, the region containing the calcium channel gene *CACNG3* (Everett et al., 2007). An association in humans has been documented between polymorphisms in *CACNA1A* (chromosome 19p13.2-p13.1) and IGE including CAE (Chioza et al., 2001). Twelve missense mutations in *CACNA1H* (chromosome 16p13.3) have been found in 14 sporadic Chinese Han patients with CAE but not in any of 230 unrelated controls (Chen et al., 2003). Mutations in three GABA receptor genes have been identified in families with CAE (sometimes in conjunction with other seizure types): *GABRG2* (Wallace et al., 2001); *GABRA1* (Maljevic et al., 2006); *GABRB3* (Feucht et al., 1999; Tanaka et al., 2008).

Four mouse models of spike-wave epilepsy are caused by mutations in genes for different subunits of voltage-gated calcium channels (VGCCs): tottering *tg*, *Cacna1a* (Fletcher et al., 1996); lethargic *lh*, *Cacnb4* (Burgess et al., 1997); stargazer *stg*, *CACNG2* (Letts et al., 1998); ducky *du*, *Cacna2d2* (Barclay et al., 2001). The *du* mutation is a genomic rearrangement resulting in the introduction of a premature stop codon and predicting the expression of a truncated protein encoded by the first three exons of *Cacna2d2*, followed by 8 novel amino acids.

This body of evidence indicates that the majority of idiopathic human and animal epilepsies are channelopathies, with implicated genes likely to be either ion channel genes or genes for related molecules (see for review Weber and Lerche, 2008).

The aim of this work was to identify disease-predisposing loci by undertaking a genome-wide SNP-based linkage scan using nuclear pedigrees which had at least two affected members (a proband with CAE and at least one other non-parental relative with CAE or JAE). A SNP-based approach provides increased genomic coverage and overall provided a greater information content in comparison to microsatellites (Evans and Cardon, 2004; John et al., 2004). A “model-free” non-parametric analysis was initially performed followed by specific parametric analyses. Fine-mapping of candidate genes was performed using a tagSNP approach, combined with re-sequencing of the coding regions and regulatory regions.

We report here the identification of a susceptibility locus for CAE on chromosome 3 and further analysis of two positional and functional candidate genes, *TRAK1* and *CACNA2D2*. Evidence for disease association was shown for variants in *TRAK1* and a putative causal variant has been investigated.

## Materials and methods

### Subjects and samples

63 nuclear pedigrees (with a total of 323 individuals; 133 CAE and 7 JAE cases) and 296 trios (affected child and both parents) were ascertained from populations with European ancestry including the UK, France, Germany, Austria, Greece, The Netherlands, Denmark, Sweden, Finland, Italy and North America. Appropriate informed consent was obtained from all participants. Full protocol and consent form approval was obtained from local research ethics committees and/or participating institutions as appropriate.

Details of the clinical data on subjects categorised as affected have been published previously (Everett et al., 2007). Inclusion criteria for CAE, based on the ILAE classification, were as follows: age of onset between 3 and 10 years; normal neurological state and development; brief and often frequent absence seizures with abrupt and severe impairment of consciousness; automatisms may occur; mild myoclonic jerks of the eyes, eyebrows or eyelids may occur; generalised tonic–clonic seizures may occur; seizures may persist into adulthood; the ictal EEG shows bilateral, synchronous, symmetrical discharges of 2.5–4 Hz spike-wave complexes on a normal background or polyspike-wave complexes; photosensitivity may be present. Exclusion criteria included: significant developmental delay; persistent or focal neurological deficit; clear abnormalities on neuroimaging. Inclusion criteria for JAE were as follows: age of onset between 10 and 17 years; normal neurological state and development; brief absence seizures with abrupt and severe impairment of consciousness occurring sporadically; generalised tonic–clonic seizures may occur possibly as the initial seizure type; mild myoclonic jerks may occur; ictal EEG of symmetrical, generalised spike-wave discharges more prominent in the frontal region at a frequency of 3.5–4.5 Hz; photosensitivity may occur. Exclusion criteria include prominent bilateral myoclonic seizures as occur in juvenile myoclonic epilepsy.

Genomic DNA was extracted from cheek swab, saliva or blood samples according to standard kits and protocols (Oragene™ and Simhelix DNA Isolation kits). 237 of the 304 samples used in the genome-wide screen were whole genome amplified (WGA) as required using the REPLI-g^®^ kit.

Control samples were obtained from the European Collection of Cell Cultures (ECACC) human random control panels 1, 2 and 3 (HRC-1, HRC-2 and HRC-3; 94 samples per plate). The ECACC HRC panels include DNA from blood donors who are all UK Caucasians and are characterised by gender and age at venesection. All donors have given written informed consent for their blood to be used for research purposes. Conformation to Hardy–Weinberg Equilibrium (HWE) was verified in all SNPs typed in controls.

### Genotyping and linkage analysis

Data for linkage analysis was obtained from a successful genome-wide screen in 240 samples from 41 of the original nuclear pedigrees (see Supplementary Table 1 for details of these 41 families). A high failure rate in WGA samples accounts for this loss of data. The screen was performed using the Illumina Linkage V Panel of 6018 SNPs. Illumina provide a GenCall score for every genotype. This score is a quality metric that indicates reliability of the genotypes called with a maximum of 1. Quality control of the data was performed using the GenCall score; a median score of 0.7 was used as the minimum acceptable value for a SNP to be used and the locus had to have been successfully typed in both genomic and WGA samples to be included in analysis. A minimal call rate of 90% was used leaving 4852 SNPs to be analysed. The average genetic distance between these SNPs was 0.77 cM and the average physical distance was 582 kb. There were no significant gaps on any chromosome.

Multipoint parametric and non-parametric linkage analyses were performed using the program MERLIN 1.1.1 (Abecasis et al., 2002) within the analysis suite EasyLinkage v5.08 (Hoffmann and Lindner, 2005). MERLIN 1.1.1 checks for and removes erroneous genotypes prior to analysis. Non-parametric Zlr scores were calculated using the Kong and Cox model (Kong and Cox, 1997) at each SNP loci, along with its corresponding *p*-value. The score is compared to a normal distribution to test for deviation. The assumption of a normal distribution is conservative as are the associated *p*-values (Whittemore and Halpern, 1994). Thresholds for suggestive evidence for linkage and significant evidence for linkage were based on those suggested by Lander and Kruglyak (1995) and are closely comparable. Suggestive linkage is defined by a *p*-value < 10^−3^ and the threshold for significant linkage is a *p*-value < 10^−4^. These values are equivalent to one-tailed *Z* scores of 3.1 and 3.7 respectively, which are themselves equivalent to maximised LOD scores of 2.4 and 3.3, which are the values suggested for genome-wide linkage analysis. Parametric analysis was performed on those genomic regions which showed significant evidence for linkage using NPL analysis; autosomal dominant inheritance with a penetrance of 50% was assumed. These parameters correspond to the available data on Mendelian segregation in this trait. They are conservative and the low penetrance reduces the risk of false negative results. A disease allele frequency of 0.01 and a phenocopy rate of 0.0001 were assumed. These values are compatible with the observed population prevalence and sibling recurrence risk ratio attributable to the locus, based on the original calculations of Risch (1990). HLOD scores as well as an estimate of *α*, which represents the proportion of pedigrees consistent with linkage at a specific locus, were calculated.

### Candidate gene analysis

Knowledge of the underlying pathophysiology of epilepsy is sufficiently advanced to allow evaluation of positional candidate genes within the critical regions according to their expression and putative function. Information was collected from a number of human genome resources including the Ensembl, National Center for Biotechnology Information (NCBI) and University of California-Santa Cruz (UCSC) Browsers (genome build NCBI36.1), in addition to the PubMed and Unigene databases. By searching through these databases, we identified the most probable functional candidate genes.

All coding and UTR exons, as well as exon–intron junctions (sequencing 100 bp either side of each exon), in the best functional candidates, were re-sequenced in 48 affected individuals from the multiple case pedigrees, using one case from each pedigree. This re-sequencing work was performed by Polymorphic DNA Technologies Inc., using standard Sanger dideoxy sequencing protocols (http://www.polymorphicdna.com).

Concurrent to the re-sequencing, tagSNPs were designed to achieve full haplotype coverage of the best functional candidates. The Tagger program (de Bakker et al., 2005) was run to identify tagSNPs. The program, using the degree of pair-wise linkage disequilibrium (LD) as measured by a *r*^2^ ≥ 0.8 between unassayed and assayed SNPs in the HapMap (release 21 CEU genotype data), identified the SNPs which would capture > 99% of all haplotypes. These tagSNPs were typed in the entire resource of nuclear pedigrees and trios and the genotypes were used to construct LD blocks with Haploview 3.2 (Barrett et al., 2005). Blocks were defined as a solid-spine of LD, i.e. the first and last marker in a block are in strong LD with all intermediate markers (one slight mismatch is allowed by the programme), but these intermediate markers are not necessarily in LD with one another. A minimum *D*′ of 0.7 was used as the cut-off point for strong LD. Intrafamilial association analysis was performed on individual SNPs using the PDT (Martin et al., 2000, 2001, 2003). The PDT produces two measures of association, the PDT-AVE and the PDT-SUM. The former gives all families equal weight in the analysis whereas the latter gives more weight to more informative families.

The same sets of tagSNPs were also genotyped in 282 ECACC controls and case–control analysis performed using the Pearson's chi-squared test or the Fisher Exact Test (when any of the cells in the calculation table contained a value of 5 or less).

Association analysis of the tagSNPs allowed the identification of gene regions for re-sequencing which would not have been re-sequenced initially, e.g. intronic areas. The limits for re-sequencing were determined by the LD structure of the tagSNPs, i.e. re-sequencing was performed around any associated SNP as far as the first SNPs either side to which it was not in LD. This re-sequencing was performed in the same 48 cases as exonic re-sequencing was performed in, by Polymorphic DNA Technologies.

Potential functional effects of identified variants were assessed using *in silico* methods, such as VisualSNP and FastSNP (Yuan et al., 2006). Standard nucleotide–nucleotide and protein–protein BLAST searches were performed to assess the degree of conservation across species and therefore to further assess how important any variant may be functionally.

## Results

### Linkage analysis

**Figure 1:**
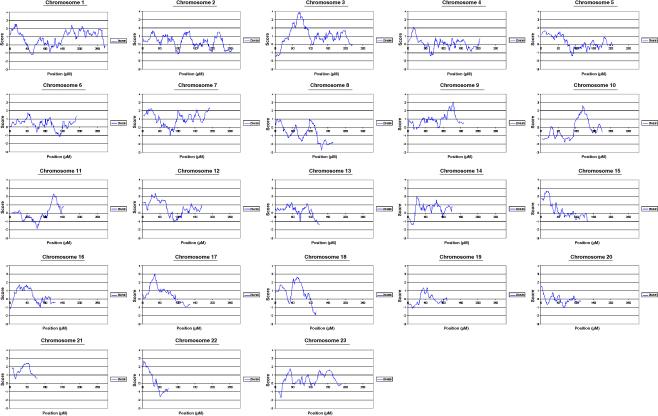
Multipoint NPL Z scores for all chromosomes. Position refers to genetic distance from the telomere of the short arm of the chromosome.

**Figure 2:**
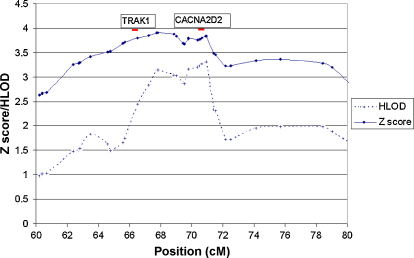
NPL *Z* score and parametric HLOD plots for chromosome 3 for the region showing evidence for linkage; position refers to genetic distance from the telomere of the short arm of the chromosome. Each marked point corresponds to a genotyped SNP. Chromosome 3 was analysed under the assumption of autosomal dominant inheritance, assuming a disease allele frequency of 0.01 and a reduced penetrance of 0.5. Also shown, with red lines, are the approximate positions of two candidate genes, *TRAK1* and *CACNA2D2*.

The results of the multipoint NPL analysis for each chromosome are shown in Fig. 1. Significant evidence for linkage was found to chromosome 3p22-p14 (NPL *Z*_max_ of 3.9, *p* < 0.0001). The critical region is arbitrarily defined as extending to where the *Z* score falls below 3; a region of approximately 20 cM (Supplementary Table 2). This is a conservative approach to defining the critical region so that no possible candidate genes are overlooked; this region contains 381 genes. Approximately 72% of families, based on per family *Z* score, were consistent with linkage to chromosome 3. An expanded view of the linked region is shown in Fig. 2.

Parametric analysis of chromosome 3 demonstrated significant evidence for linkage with a maximum HLOD (HLOD_max_) of 3.3 (*α* = 0.7) occurring at 3p21 (Fig. 2).

### Candidate gene analysis

Based on reported function, expression patterns and animal models, we identified two highly plausible functional candidate genes within the chromosome 3p22-p14 linkage region: *TRAK1* and *CACNA2D2*.

**Figure 3:**
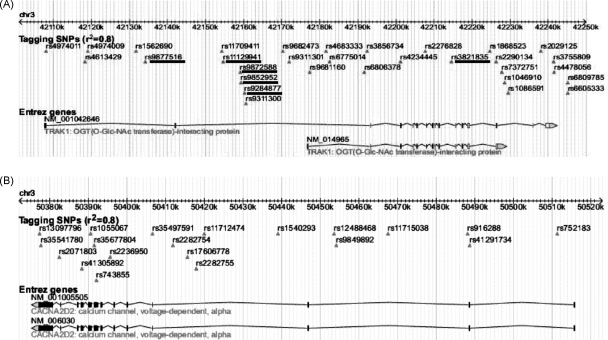
(a and b) TagSNPs for *TRAK1* and *CACNA2D2*. Underlined SNPs demonstrated evidence for transmission disequilibrium with intra-familial association analysis using the PDT.

Twenty tagSNPs were typed in *CACNA2D2*; 31 tagSNPs were typed in *TRAK1*. These were selected to encompass the full possible genomic region covered by the reported transcripts in the NCBI database (Fig. 3a and b).

Intrafamilial and case–control association analysis of the *CACNA2D2* tagSNPs did not detect any evidence for overtransmission of any alleles therefore *CACNA2D2* was not analysed further.

**Figure 4:**
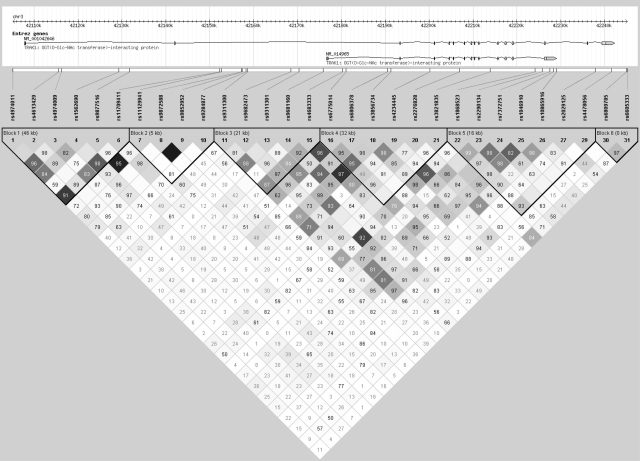
LD structure of *TRAK1* based on Haploview analysis of the whole resource. Strong LD is defined as *D*′ > 0.8. Values for pairwise *D*′ (as a percentage) are given in each square except for those where *D*′ = 1 in which case the square is blank. Level of *r*^2^ LD is indicated by the colour of the square; the darker the square, the higher the pairwise *r*^2^ value.

**Figure 5:**
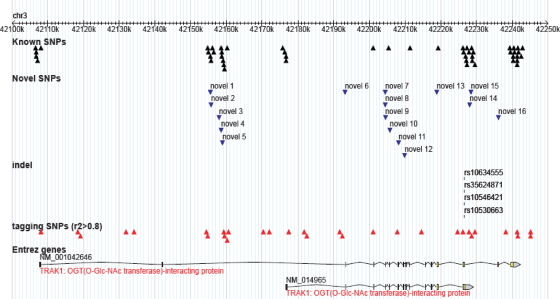
Variants found via re-sequencing in *TRAK1*. “Known SNPs” are those which have previously been identified and can be found in the dbSNP database. “Novel SNPs” are those which have not previously been identified. The “indel” corresponds to the triplet repeat.

Intrafamilial association analysis on the entire resource detected evidence for overtransmission of the minor allele to affected offspring in two SNPs in *TRAK1* (at the 95% significance level; Table 1). The LD block structure of *TRAK1* as predicted using Haploview is shown in Fig. 4. Both disease-associated SNPs reside in block 2, a 5 kb region of high LD; therefore it was decided to re-sequence this region at the same time as the coding regions as part of the re-sequencing effort. Re-sequencing of this so-called “associated block” was extended to the two flanking SNPs (rs11709411 and rs9311300; see Figs. 3a and 4) in order to capture all variants that might be in LD with the associated SNPs in block 2. Re-sequencing of this region (∼5.5 kb) and the coding exons (and 100 bp either side), the 5′ and 3′ UTR regions, and 1 kb upstream and downstream of the gene (a total of ∼23.5 kb) identified 51 known variants and 16 novel variants (Supplementary Table 3 and Fig. 5). Based on bioinformatics analysis, the variant in *TRAK1* which currently seemed to be the most likely to have a functional effect was a triplet repeat in the final exon of the shorter transcript NM_014965. The repeat inserts additional glutamic acid residues in a run of glutamic acid residues and has been reported in the NCBI SNP database as several different indels; rs10634555, rs35624871, rs10546421 and rs10530663. The repeat was therefore genotyped in 96 ECCAC samples to ascertain the number of alleles and their frequency, and in all the individuals of the 63 pedigrees to determine any significant association. The ECCAC sample genotyping showed four alleles with frequencies: 0.23, 0.24, 0.23 and 0.30. In the 63 probands from the pedigrees, the comparative frequencies were: 0.23, 0.29, 0.27 and 0.22. However, the results of the case–control and intrafamilial association analyses were not statistically significant.

## Discussion

Childhood absence epilepsy (CAE) is an idiopathic generalised epilepsy (IGE) with a well defined and homogeneous phenotype that displays a clear electrophysiological hallmark and clear evidence of a genetic aetiology. The clinical criteria adopted here provide the reasonable expectation that the patients ascertained represent a homogenous clinical phenotype. It is has been shown that IGE phenotypes may cluster in families with a proband with absence epilepsy, but analysis reveals an increased clustering of CAE and JAE (Berkovic et al., 1987; Marini et al., 2004) suggesting that they may share susceptibility loci. For this reason, pedigrees in which first degree relatives of a proband with CAE had a diagnosis of JAE were included and such individuals were categorised as affected (6 of the 63 pedigrees).

We have identified a novel susceptibility locus for CAE/JAE on chromosome 3p23-p14 through a SNP-based genome-wide linkage scan (*Z*_mean_ = 3.9, *p* < 0.0001; HLOD = 3.3, *α* = 0.7). This is the only region of the genome for which there is a significant linkage score in our analysis. The linkage peaks on chromosomes 9 and 17 which are close to being statistically suggestive of linkage might warrant some attention in future in context of the genetically complex architecture of IGE syndromes.

The parametric analysis of chromosome 3 indicates that a large proportion of our pedigrees (∼70%) are consistent with linkage to chromosome 3p23-p14. We have previously demonstrated evidence for linkage to 16p12-p13.1, a region containing the calcium channel gene *CACNG3*, using a similar resource of CAE/JAE families (Everett et al., 2007). CAE is a complex trait and therefore we expect significant locus heterogeneity, with it being quite possible that any family or individual may have a contribution from more than one locus. Twenty-one pedigrees are consistent with linkage to both the 16p12-p13.1 locus and the 3p23-p14 locus identified in the current study. It is possible that this is suggestive of interaction between two loci culminating in increased seizure susceptibility. There is some evidence from mouse models that different genes can interact and alter seizure susceptibility. For example, a combination of mutant alleles in *Scn2a* and *Kcnq2* resulted in a much more severe epilepsy phenotype than when mutations were only present in a single gene (Kearney et al., 2006). This is not something which we can currently directly test as it requires identification of unambiguous mutations.

Data-mining of this linked region identified *TRAK1* (trafficking protein, kinesin binding) and *CACNA2D2* (calcium channel, voltage dependent, alpha 2/delta subunit 2) as the most plausible functional candidate genes. Both genes were investigated with tagSNPs but only variants in *TRAK1* demonstrated significant evidence for transmission disequilibrium. This does not however definitively exclude *CACNA2D2* as tagSNPs will only detect disease-associated alleles present at a comparable frequency. It is possible that rare variants present in a subset of the study population will not be detected using this methodology.

*TRAK1* encodes a trafficking protein originally known as OIP106 that was initially identified as a binding partner for the enzyme β-O-linked N-acetylglucosamine (O-GlcNAc) transferase (Iyer et al., 2003). A homozygous frameshift mutation in the mouse Trak1 gene, which produces a truncated protein, was found to cause a recessively transmitted form of hypertonia, a neurological dysfunction characterised by postural abnormalities, jerky movements, and tremor (Gilbert et al., 2006). Altered levels of γ-amino-*n*-butyric acid A (GABA_A_) receptors observed in Trak1 mutant mice suggested that Trak1 may also participate in the regulation of GABA_A_ receptor endosomal trafficking. The protein has been shown to interact with the GABA_A_ receptor α1 subunit and it has been hypothesised that Trak1 has a crucial role in regulating the endocytic trafficking of these receptors and may facilitate the targeting of endocytosed GABA_A_ receptors back to the cell surface or block them from degradation (Webber et al., 2008). In the mammalian central nervous system, GABA is the main inhibitory neurotransmitter, activating GABA_A_ receptors (GABA_A_Rs) on target neurones either in a phasic or a tonic fashion (Farrant and Nusser, 2005; Mody and Pearce, 2004). GABA_A_ receptors have been repeatedly documented to play a critical role in animal models of seizures. The initial GABA_A_ receptor mutations associated with the idiopathic generalised epilepsies were found in the γ2 and α1 subunits, consistent with a genetic defect in phasic, inhibitory GABAergic synaptic inhibition.

Intrafamilial association analysis identified two tagSNPs showing evidence for transmission disequilibrium indicating disease association. However, re-sequencing of the surrounding region, which includes exon 2 of isoform 1, did not identify any putative causal variants. There are three main explanations for this outcome. It is possible that the association results may both be false positives; although this is unlikely as intrafamilial association analysis is robust to artefacts such as population substructure. However, a Bonferroni style correction for multiple testing was not applied to these analyses because although this would reduce the risk of type I errors occurring (detection of a false positive), it would simultaneously have increased the risk of type II errors (missing any true associations). Thus the association evidence described must be considered as tentative but requiring independent replication.

A second explanation is that the associated tagSNPs may be in long-range LD with causal variants not identified by the re-sequencing effort (Lawrence et al., 2005). Finally, it is possible that the two associated tagSNPs are themselves causal. It remains very difficult to predict the effect of intronic variants but that does not mean that they do not have an effect; it has, for example, been shown that a common intronic variant in the chemokine receptor gene *CXCR3* is associated with gene expression levels and asthma risk (Choi et al., 2008).

In conclusion, these observations provide genetic evidence of a novel susceptibility locus for childhood absence epilepsy on chromosome 3p23-p14. Common variants showing transmission disequilibrium have been identified in the candidate gene *TRAK1*. Definitive evidence to confirm or exclude this gene will require re-sequencing across an extended genomic region encompassing *TRAK1* in a larger number of patients and a better understanding of the role that non-exonic SNPs play in regulation. We have identified a large number of novel intronic and 3′ UTR SNPs, the possible function of which it is currently extremely difficult to predict. The latter, in particular, may be located within target sites for miRNA molecules, but information is lacking in this area and it is not something which we have assessed in detail. Replication studies in similar resources of CAE patients are required, and future studies into the biological mechanisms of CAE could suggest other candidate genes in the linked region which would need to be re-sequenced to identify putative causal variants.

## Figures and Tables

**Table 1:** Summary of those SNPs in *TRAK1* which demonstrated significant evidence (*p* < 0.05 in at least one test statistic) for overtransmission of the minor allele in the entire resource of 63 pedigrees and 296 trios.

SNP	Number transmitted: number not transmitted	Sum-PDT $\chi_{\left(1\text{df}\right)}^{2}$	*Z*-score	*p*-Value	Ave-PDT $\chi_{\left(1\text{df}\right)}^{2}$	*Z*-score	*p*-Value
rs9872588	65:41	6.88	2.62	0.009	5.48	2.34	0.019
rs9852952	59:40	5.69	2.39	0.017	4.17	2.04	0.041
