# Supplementary material for: Genome wide high density SNP-based linkage analysis of childhood absence epilepsy identifies a susceptibility locus on chromosome 3p23-p14
Source: Epilepsy Res. 2009 Dec;87(2-3):247–55. doi: 10.1016/j.eplepsyres.2009.09.010 (PMC2791882; doi:10.1016/j.eplepsyres.2009.09.010)
Supplement: Supplementary file 1 [file mmc1.doc]

Supplementary Table 1: Summary family information for the 41 pedigrees included in the genome screen.

| **Pedigree number** | **Number of affected individuals in pedigree** | **Type of epilepsy (or seizure)** |
| --- | --- | --- |
| 5 | 2 (sister and brother) | Sister:CAE  Brother:CAE |
| 26 | 2 (brother and sister) | Brother:CAE (with FS)  Sister:CAE (with FS) |
| 27 | 3 (mother, her daughter and son) | Mother:CAE (with GTCS)  Daughter:CAE (with GTCS)  Son:CAE |
| 92 | 2 (two sisters) | Sister #1:CAE  Sister #2:CAE |
| 96 | 2 (two sisters) | Sister #1:CAE  Sister #2:CAE |
| 173 | 2 (two sisters) | Sister #1:CAE  Sister #2:CAE |
| 202 | 4 (father, his two sons and a daughter) | Father:CAE (with FS)  Son #1:CAE (with FS)  Son #2:CAE (with FS)  Daughter:CAE (with FS) |
| 317 | 2 (sister and brother) | Sister:CAE (with GTCS)  Brother:CAE |
| 321 | 4 (father and three daughters) | Father:CAE (with GTCS)  Daughter #1:CAE  Daughter #2:CAE  Daughter #3:CAE (with FS) |
| 346 | 2 (two sisters) | Sister #1:CAE  Sister #2:CAE |
| 348 | 2 (2 brothers) | Brother #1:CAE  Brother #2:CAE |
| 353 | 3 (father and two affected sons) | Father:CAE  Son #1:CAE  Son #2:CAE |
| 393 | 2 (brother and sister) | Brother:CAE  Sister:CAE (with GTCS) |
| 395 | 2 (brother and sister) | Brother:CAE  Sister:CAE |
| 396 | 3 (father, his son and daughter) | Father:CAE  Son:CAE  Daughter:CAE |
| 400 | 2 (brother and sister) | Brother:CAE (with GTCS)  Sister:CAE (with GTCS) |
| 401 | 3 (father, his son and daughter) | Father:CAE (with GTCS)  Son:CAE (with GTCS)  Daughter:CAE (with GTCS) |
| 403 | 2 (sister and brother) | Sister:CAE (with GTCS)  Brother:CAE (with FS) |
| 404 | 5 (mother, her daughter and two sons, maternal grandfather) | Maternal grandfather:CAE (with GTCS)  Mother:CAE (with GTCS)  Daughter:CAE  Son #1:CAE  Son #2:CAE |
| 407 | 3 (mother and her two daughters) | Mother:CAE  Daughter #1:CAE  Daughter #2:CAE |
| 421 | 2 (two sisters) | Sister #1:CAE  Sister #2:CAE |
| 422 | 3 (sister and brother and their maternal uncle) | Maternal uncle:CAE (with GTCS)  Sister:CAE  Brother:CAE |
| 441 | 2 (sister and brother) | Sister:CAE  Brother:JAE |
| 443 | 2 (two sisters) | Sister #1:CAE  Sister #2:CAE |
| 445 | 2 (brother and sister) | Brother:CAE (with FS)  Sister:CAE |
| 446 | 2 (two brothers) | Brother #1:CAE  Brother #2:CAE |
| 447 | 2 (two sisters) | Sister #1:CAE  Sister #2:CAE |
| 461 | 2 (sister and brother) | Sister:CAE  Brother:CAE |
| 466 | 2 (two sisters) | Sister #1:CAE  Sister #2:CAE |
| 527 | 2 (two sisters) | Sister #1:CAE  Sister #2:CAE |
| 577 | 2 (brother and sister) | Brother:CAE  Sister:CAE |
| 586 | 3 (mother, her daughter and son) | Mother:CAE  Daughter:CAE  Son:CAE |
| 871 | 2 (two sisters) | Sister #1:CAE  Sister #2:CAE |
| 872 | 2 (brother and sister) | Brother:CAE  Sister:CAE |
| 876 | 2 (brother and sister) | Brother:CAE  Sister:CAE (with GTCS) |
| 878 | 2 (two brothers) | Brother #1:CAE  Brother #2:CAE |
| 879 | 3 (mother and daughter and the latter’s maternal aunt) | Aunt:CAE (with FS)  Mother:CAE (with FS)  Daughter:CAE |
| 880 | 2 (two sisters) | Sister #1:CAE  Sister #2:CAE (with FS and GTCS) |
| 881 | 3 (two brothers and a sister) | Brother #1:CAE (with FS)  Brother #2:CAE (with GTCS)  Sister:CAE |
| 884 | 3 (mother and her two daughters) | Mother:CAE (with GTCS)  Daughter #1:CAE (with GTCS)  Daughter #2:CAE |

Supplementary Table 2: Linkage-Analysis Data for the Critical Region on Chromosome 3

| **SNP** | **Physical Position (Mb)** | **Genetic Position (cM)** | **Z Score** | **P value** |
| --- | --- | --- | --- | --- |
| rs1455326 | 35417126 | 60.669 | 2.69 | 0.004 |
| rs967672 | 36801595 | 62.383 | 3.25 | 0.0006 |
| rs1392748 | 37240317 | 62.721 | 3.28 | 0.0005 |
| rs883523 | 37286096 | 62.723 | 3.28 | 0.0005 |
| rs267538 | 37560743 | 62.806 | 3.3 | 0.0005 |
| rs762318 | 38491373 | 63.501 | 3.41 | 0.0003 |
| rs1405796 | 39669773 | 64.588 | 3.51 | 0.0002 |
| rs1996562 | 40110750 | 64.750 | 3.52 | 0.0002 |
| rs749932 | 41353625 | 65.561 | 3.69 | 0.00011 |
| rs1495704 | 41478316 | 65.682 | 3.71 | 0.0001 |
| rs477078 | 42345598 | 66.521 | 3.79 | 0.00008 |
| rs13061576 | 42807983 | 67.235 | 3.85 | 0.00006 |
| rs737516 | 43533089 | 67.730 | 3.9 | 0.00005 |
| rs1013758 | 43601379 | 67.810 | 3.9 | 0.00005 |
| rs954282 | 44728367 | 68.843 | 3.87 | 0.00006 |
| rs2056321 | 45058137 | 69.017 | 3.84 | 0.00006 |
| rs1860264 | 45898089 | 69.459 | 3.69 | 0.00011 |
| rs737452 | 46002414 | 69.514 | 3.67 | 0.00012 |
| rs1520483 | 46485213 | 69.768 | 3.78 | 0.00008 |
| rs1402152 | 46549422 | 69.802 | 3.79 | 0.00007 |
| rs1014228 | 47627643 | 70.369 | 3.75 | 0.00009 |
| rs319682 | 47890122 | 70.507 | 3.77 | 0.00008 |
| rs1865741 | 49337896 | 70.655 | 3.8 | 0.00007 |
| rs7061 | 50089519 | 70.891 | 3.83 | 0.00006 |
| rs2236947 | 50346436 | 70.893 | 3.83 | 0.00006 |
| rs7433217 | 50962234 | 70.894 | 3.83 | 0.00006 |
| rs11720298 | 51556549 | 70.941 | 3.83 | 0.00006 |
| rs1133415 | 52550871 | 71.391 | 3.49 | 0.0002 |
| rs11235 | 52720127 | 71.535 | 3.46 | 0.0003 |
| rs2101397 | 53473982 | 72.173 | 3.22 | 0.0006 |
| rs893367 | 53884771 | 72.521 | 3.22 | 0.0006 |
| rs9864433 | 54570718 | 74.123 | 3.34 | 0.0004 |
| rs920891 | 55246276 | 75.709 | 3.36 | 0.0004 |
| rs536036 | 57301923 | 78.436 | 3.29 | 0.0005 |
| rs1444185 | 58662017 | 79.016 | 3.2 | 0.0007 |
| rs1472653 | 59560632 | 80.094 | 2.89 | 0.002 |

Supplementary Table 3. Summary of those SNPs found in the re-sequencing of 48 affected individuals from families consistent with linkage.

| **dbSNP ID** | **Gene Position**  **(NM_001042646/NM_014965)** | **HGVS Name or variant type** |
| --- | --- | --- |
| rs1995136 | 5’ near gene/- | NT_022517.17:g.42071505G>C |
| rs2173336 | 5’ near gene/- | NT_022517.17:g.42071738C>T |
| rs2133069 | 5’ near gene/- | NT_022517.17:g.42071833C>G |
| rs4974012 | 5’ near gene/- | NT_022517.17:g.42072043A>G |
| rs4974011 | intron 1/- | NM_001042646.1:c.91+97C>G  NT_022517.17:g.42073153C>G |
| rs11129941 | intron 2/- | NM_001042646.1:c.286+12573T>C  NT_022517.17:g.42119683T>C |
| rs9311297 | intron 2/- | NM_001042646.1:c.286+12764G>A  NT_022517.17:g.42119874G>A |
| rs4974007 | intron 2/- | NM_001042646.1:c.286+13340A>G  NT_022517.17:g.42120450A>G |
| novel 1 | intron 2/- | NM_001042646.1:c.286+13373G>A  NT_022517.17:g.42120483G>A |
| rs2055285 | intron 2/- | NM_001042646.1:c.286+13545T>C  NT_022517.17:g.42120655T>C |
| novel 2 | intron 2/- | NM_001042646.1:c.286+13586A>T  NT_022517.17:g.42120696A>T |
| rs1874756 | intron 2/- | NM_001042646.1:c.286+13933A>G  NT_022517.17:g.42121043A>G |
| rs11129942 | intron 2/- | NM_001042646.1:c.286+14290C>T  NT_022517.17:g.42121400C>T |
| novel 3 | intron 2/- | NM_001042646.1:c.286+15874G>C  NT_022517.17:g.42122984G>C |
| rs9311298 | intron 2/- | NM_001042646.1:c.286+16347A>C  NT_022517.17:g.42123457A>C |
| novel 4 | intron 2/- | NM_001042646.1:c.286+16457G>A  NT_022517.17:g.42123567G>A |
| rs9851827 | intron 2/- | NM_001042646.1:c.286+16586C>G  NT_022517.17:g.42123696C>G |
| rs9852262 | intron 2/- | NM_001042646.1:c.286+16670G>C  NT_022517.17:g.42123780G>C |
| novel 5 | intron 2/- | NM_001042646.1:c.286+16678G>A  NT_022517.17:g.42123788G>A |
| rs9852329 | intron 2/- | NM_001042646.1:c.286+16895C>T  NT_022517.17:g.42124005C>T |
| rs9872588 | intron 2/- | NM_001042646.1:c.286+17067T>G  NT_022517.17:g.42124177T>G |
| rs9852952 | intron 2/- | NM_001042646.1:c.286+17255C>T  NT_022517.17:g.42124365C>T |
| rs9284877 | intron 2/- | NM_001042646.1:c.286+18029C>T  NT_022517.17:g.42125139C>T |
| rs9311302 | intron 2/5’ near gene | NM_001042646.1:c.287-17612T>C  NT_022517.17:g.42140698T>C |
| rs9311303 | intron 2/5’ near gene | NM_001042646.1:c.287-17069T>C  NT_022517.17:g.42141241T>C |
| rs9757367 | intron 2/5’ near gene | NM_001042646.1:c.287-16697A>G  NT_022517.17:g.42141613A>G |
| rs9755034 | intron 2/5’ UTR | NM_001042646.1:c.287-16547G>A  NM_014965.3:c.-95G>A  NT_022517.17:g.42141763G>A |
| novel 6 | intron 2/intron 1 | NM_001042646.1:c.287-64A>T  NM_014965.3:c.113-64A>T  NT_022517.17:g.42158246A>T |
| rs4234445 | intron 3/intron 2 | NM_001042646.1:c.364-26T>C  NM_014965.3:c.190-26T>C  NT_022517.17:g.42166155T>C |
| novel 7 | intron 4/intron 3 | NM_001042646.1:c.481-139A>T  NM_014965.3:c.307-139A>T  NT_022517.17:g.42169402A>T |
| novel 8 | intron 4/intron 3 | NM_001042646.1:c.481-16T>C  NM_014965.3:c.307-16T>C  NT_022517.17:g.42169525T>C |
| novel 9 | intron 4/intron 3 | NM_001042646.1:c.481-10G>T  NM_014965.3:c.307-10G>T  NT_022517.17:g.42169531G>T |
| rs3816396 | intron 5/intron 4 | NM_001042646.1:c.582-113C>T  NM_014965.3:c.408-113C>T  NT_022517.17:g.42170399C>T |
| novel 10 | intron 6/intron 5 | NM_001042646.1:c.690+39T>A  NM_014965.3:c.516+39T>A  NT_022517.17:g.42170659T>A |
| novel 11 | intron 7/intron 6 | NM_001042646.1:c.769+102G>A  NM_014965.3:c.595+102G>A  NT_022517.17:g.42173234G>A |
| novel 12 | intron 8/intron 7 | NM_001042646.1:c.900+117G>C  NM_014965.3:c.726+117G>C  NT_022517.17:g.42174818G>C |
| rs9851455 | exon 10/exon 9 | NM_001042646.1:c.1095A>G  NM_014965.3:c.921A>G  NP_001036111.1:p.S365S  NP_055780.2:p.S307S  NT_022517.17:g.42176418A>G |
| novel 13 | intron 12/intron 11 | NM_001042646.1:c.1428-61G>C  NM_014965.3:c.1254-61G>C  NT_022517.17:g.42183870G>C |
| rs9311309 | intron 13/intron 12 | NM_001042646.1:c.1744+77G>A  NM_014965.3:c.1570+77G>A  NT_022517.17:g.42184325G>A |
| rs2290134 | exon 14/exon 13 | NM_001042646.1:c.1749C>T  NM_014965.3:c.1575C>T  NP_001036111.1:p.S583S  NP_055780.2:p.S525S  NT_022517.17:g.42191266C>T |
| rs2290133 | exon 14/exon 13 | NM_001042646.1:c.1815C>T  NM_014965.3:c.1641C>T  NP_001036111.1:p.P605P  NP_055780.2:p.P547P  NT_022517.17:g.42191332C>T |
| rs3836496 | intron 14/3’ UTR | -/T indel |
| rs3196165 | intron 14/3’ UTR | NM_001042646.1:c.1963+664A>G  NM_014965.3:c.*392A>G  NT_022517.17:g.42192145A>G |
| rs9311311 | intron 14/3’ UTR | NM_001042646.1:c.1963+797G>A  NM_014965.3:c.*525G>A  NT_022517.17:g.42192278G>A |
| rs34348608 | intron 14/3’ UTR | NM_001042646.1:c.1963+884_1963+885insT  NM_014965.3:c.*612_*613insT  NT_022517.17:g.42192365_42192366insT |
| rs7372751 | intron 14/3’ UTR | NM_001042646.1:c.1963+1447G>C  NM_014965.3:c.*1175G>C  NT_022517.17:g.42192928G>C |
| novel 14 | intron 14/3’ UTR | NM_001042646.1:c.1963+1613G>A  NM_014965.3:c.*1341G>A  NT_022517.17:g.42193094G>A |
| novel 15 | intron 14/3’ UTR | NM_001042646.1:c.1963+1869C>T  NM_014965.3:c.*1597C>T  NT_022517.17:g.42193350C>T |
| rs1046910 | intron 14/3’ UTR | NM_001042646.1:c.1963+2187C>T  NM_014965.3:c.*1915C>T  NT_022517.17:g.42193668C>T |
| rs9747 | intron 14/3’ UTR | NM_001042646.1:c.1963+2256T>C  NM_014965.3:c.*1984T>C  NT_022517.17:g.42193737T>C |
| rs9511 | intron 14/3’ UTR | NM_001042646.1:c.1963+2280C>T  NM_014965.3:c.*2008C>T  NT_022517.17:g.42193761C>T |
| rs11546404 | intron 14/3’ UTR | A/C |
| rs10865916 | intron 14/3’ near gene | NM_001042646.1:c.1963+3006G>T  NT_022517.17:g.42194487G>T |
| novel 16 | intron 15/- | NM_001042646.1:c.2066+64A>G  NT_022517.17:g.42201156A>G |
| rs3733053 | intron 15/- | NM_001042646.1:c.2067-41A>G  NT_022517.17:g.42204397A>G |
| rs3733054 | intron 15/- | NM_001042646.1:c.2067-12T>C  NT_022517.17:g.42204426T>C |
| rs12487911 | exon 16/- | NM_001042646.1:c.2799T>C  NP_001036111.1:p.P933P  NT_022517.17:g.42205169T>C |
| rs3836498 | 3’ UTR/- | NM_001042646.1:c.*45_*47del3  NT_022517.17:g.42205278_42205280del3 |
| rs3774391 | 3’ UTR/- | NM_001042646.1:c.*140C>T  NT_022517.17:g.42205373C>T |
| rs1043610 | 3’ UTR/- | NM_001042646.1:c.*405C>T  NT_022517.17:g.42205638C>T |
| rs1043621 | 3’ UTR/- | NM_001042646.1:c.*1117A>G  NT_022517.17:g.42206350A>G |
| rs1043622 | 3’ UTR/- | NM_001042646.1:c.*1120C>T  NT_022517.17:g.42206353C>T |
| rs3755809 | 3’ UTR/- | NM_001042646.1:c.*1131T>C  NT_022517.17:g.42206364T>C |
| rs4478056 | 3’ UTR/- | NM_001042646.1:c.*1392T>C  NT_022517.17:g.42206625T>C |
| rs9156 | 3’ UTR/- | NM_001042646.1:c.*1604C>A  NT_022517.17:g.42206837C>A |
| rs11715575 | 3’ near gene/- | NT_022517.17:g.42207488A>G |
| rs11707951 | 3’ near gene/- | C/T |
